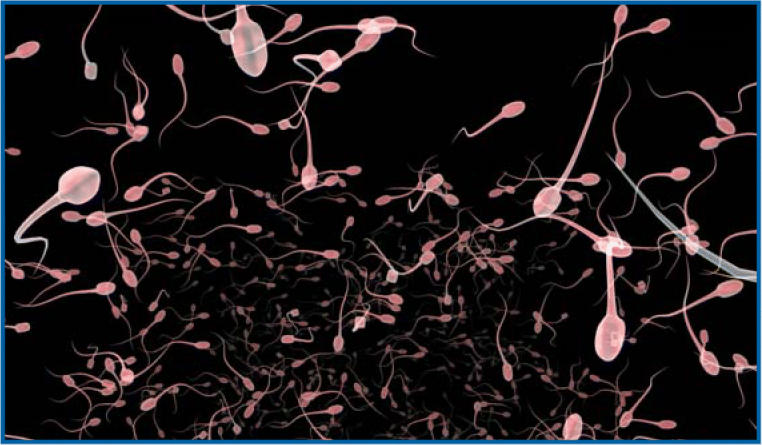# Headliners: Reproductive Health: Sperm DNA Changes as Men Age

**Published:** 2006-09

**Authors:** Jerry Phelps

Wyrobek AJ, Eskenazi B, Young S, Arnheim N, Tiemann-Boege I, Jabs EW, et al. 2006. Advancing age has differential effects on DNA damage, chromatin integrity, gene mutations, and aneuploidies in sperm. Proc Natl Acad Sci U S A 103:9601–9606.

In the past several decades, more men and women have been postponing parenthood. Fatherhood among men aged 35–49 has increased 40%, while there has been a 20% decline in births fathered by men under age 30. Although it has long been accepted that women face reproductive challenges with age, the consequences of delaying fatherhood have been less understood. Now NIEHS grantees Andrew J. Wyrobek of Lawrence Livermore National Laboratory and Brenda Eskenazi of the University of California, Berkeley, School of Public Health, with their colleagues, have produced new research that suggests that, like women, men too have a “biological clock,” but one that causes a more gradual change in fertility.

Obstetrician/gynecologists have known for quite a while that as women age, their risk of miscarriage increases, as does the risk of having children with Down syndrome or other genetic defects. Advanced paternal age has also been implicated in a range of reproductive and genetic abnormalities, from reduced fertility to some diseases of complex etiology such as schizophrenia. This research team has previously reported that as men age, their sperm counts decline and their sperm become less active. However, the mechanisms for the effect of older paternal age on genetic defects are not well understood.

In this study, the researchers analyzed the sperm of 97 men after an average of 5.1 days of sexual abstinence. The men were nonsmokers aged 22–80 who were in generally good health. The researchers used flow cytometry and statistical analysis to observe associations between a subject’s age, his semen quality, and genomic abnormalities in his sperm such as DNA fragmentation, aneuploidy, diploidy, and mutations related to achondroplasia and Apert syndrome.

Increased age of the men was not associated with the same genetic defects seen in older women; for instance, there was no increased risk of fathering a child with Down syndrome. But some older fathers did have an increased risk of having children with achondroplasia, and according to the published results, ”a small fraction of men are at increased risk for transmitting multiple genetic and chromosomal defects.” The authors caution that their findings are preliminary and are based on a small number of tests in a small population of men. However, they believe their findings suggest that as men age, they may have more difficulty fathering children.

## Figures and Tables

**Figure:**